# Cryo-EM structure of L-fucokinase/GDP-fucose pyrophosphorylase (FKP) in *Bacteroides fragilis*

**DOI:** 10.1007/s13238-018-0576-x

**Published:** 2018-09-21

**Authors:** Ying Liu, Huifang Hu, Jia Wang, Qiang Zhou, Peng Wu, Nieng Yan, Hong-Wei Wang, Jia-Wei Wu, Linfeng Sun

**Affiliations:** 1 Tsinghua-Peking Joint Center for Life Sciences, School of Life Sciences, Tsinghua University, Beijing, 100084 China; 2 School of Life Sciences, Tsinghua University, Beijing, 100084 China; 3 Department of Molecular Medicine, The Scripps Research Institute, La Jolla, CA 92037 USA; 4 Ministry of Education Key Laboratory of Protein Sciences, Beijing Advanced Innovation Center for Structural Biology, School of Life Sciences, Tsinghua University, Beijing, 100084 China; 5 State Key Laboratory of Membrane Biology, Beijing Advanced Innovation Center for Structural Biology, School of Life Sciences, Tsinghua University, Beijing, 100084 China; 6 CAS Center for Excellence in Molecular Cell Science, Hefei National Laboratory for Physical Sciences at Microscale, School of Life Sciences, University of Science and Technology of China, Hefei, 230027 China

**Dear Editor,**

L-Fucose (6-deoxy-l-galactose, fucose) is the basic component of a variety of glycan structures. The fucosylated oligosaccharides participate in a variety of cellular activities, like the cell-cell recognition, selectin-mediated leukocyte-endothelial adhesion and the formation of Lewis blood group antigens (Ma et al., 2006). GDP-fucose is an important fucose donor in the process of fucosylated oligosaccharides formation. Two pathways of GDP-fucose synthesis are present in the cytosol of mammalian cells, including the *de novo* pathway and the salvage pathway (Becker et al., 2003). In the salvage pathway, cells use fucose from the extracellular or lysosomal sources to synthesize GDP-fucose. Two enzymes catalyze the production of GDP-fucose from fucose. First, the fucokinase catalyzes the formation of fucose-1-phosphate from fucose, with ATP consumption to provide the energy (Park et al., 1998). GDP-fucose pyrophosphorylase (GFPP) then uses fucose-1-phosphate and GTP to synthesize GDP-fucose (Pastuszak et al., 1998). In bacteria and plants, the two independent catalytic reactions in the salvage pathway can be accomplished by a single enzyme, the fucokinase/GDP-fucose pyrophosphorylase (FKP) (Coyne et al., 2005; Kotake et al., 2008). FKP can convert l-fucose into GDP-fucose *via* a fucose-1-phosphate (Fuc-1-P) intermediate (Fig. 1A), and has been applied to the chemical synthesis of fucose-containing glycans and glycoconjugates (Wang et al., 2009; Yu et al., 2012). Due to the importance of FKP in the biosynthesis of fucosylated polysaccharides, efforts have been put into the structural investigations of FKP (Quirk, 2012; Cheng et al., 2014). However, the three dimensional architecture of FKP remains to be elusive.

**Figure 1:**
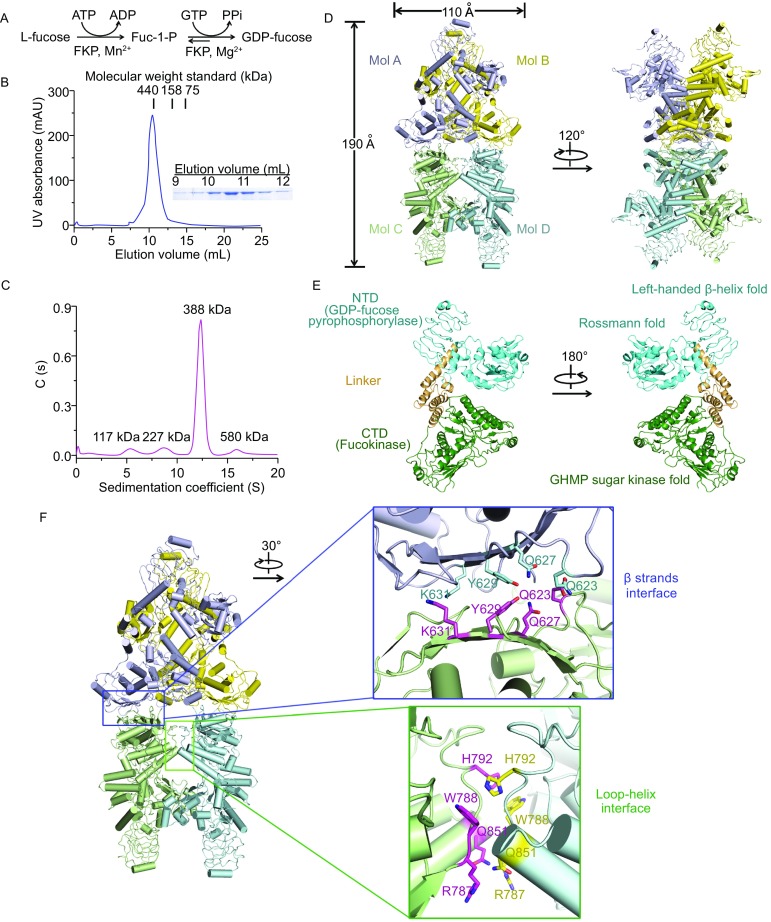
**Overall structure of FKP from** ***Bacteroides fragilis***. (A) Enzymatic scheme of GDP-fucose synthesis by FKP. (B) The wild type FKP forms a tetramer on the size exclusion chromatography (SEC). (C) Analytical ultracentrifugation analysis (AUC) of the wild type FKP. (D) Overall structure of the FKP tetramer in two views. The four protomers (A–D) are colored blue, yellow, green and cyan, respectively. (E) Structure of the FKP protomer. The NTD, CTD and the linker in between are colored cyan, green and orange, respectively. The NTD contains a Rossmann fold and a left-handed β-helix fold. The CTD contains a GHMP sugar kinase fold. Two perpendicular views are shown. All structural figures were prepared using PyMol. (F) FKP tetramer interfaces. Two interfaces are identified between FKP subunits. One is mediated by the β strands and the other is mediated by the loop-helix. Close-up views of the key residues involved in the interfaces are shown on the right

We overexpressed and purified the full-length wild type FKP from *Bacteroides fragilis* 9343. The calculated molecular weight of FKP is ~ 105 kDa. When applied to size exclusion chromatography, FKP was eluted at a volume corresponding to that of the standard molecular weight marker of 440 kDa, indicating that FKP may form an oligomer in solution, possibly a tetramer (Fig. 1B). Further characterization of the purified FKP with analytical ultracentrifugation sedimentation velocity (AUC-SV) revealed a dominant sedimentation coefficient with a molecular weight of 338 kDa (Fig. 1C).

Next, we applied the purified FKP protein to cryo-EM single particle analysis. The most homogeneous particles were selected after several rounds of 2D and 3D classifications, and applied to 3D auto-refinement. An electron density map with an overall resolution of 4.2 Å was achieved by applying D2 symmetry. To further improve the resolution, we applied a local mask for the FKP CTD during 3D auto-refinement. The resolution of this region was improved to 3.9 Å (Fig. S1). For the highly flexible NTD, we expanded the 96,749 particles by four-fold based on the tetrameric organization and D2 symmetry. These particles were first rotated to the same position for NTD and then classified without alignment with a local mask. Through C1 symmetry auto-refinement, the final reconstruction of these particles greatly improved the map quality for NTD. In the end, we got a cyro-EM structure of the full-length FKP at 4.0 Å resolution with a C1 symmetry (Fig. S1B). Further details related to data processing are summarized in Table S1. Homologous structures were used to build the structure model for the NTD and CTD of FKP. Crystal structures of the d-glycero-d-manno-heptose 1-phosphate kinase from *Bacteriodes thetaiotaomicron* (PDB code: 3K85) and the potato tuber ADP-glucose pyrophosphorylase (Jin et al., 2005, PDB code: 1YP3) can be docked well into the CTD and NTD of FKP, respectively, as a rigid body. These structures were used as initial models for further refinement. The final structure model contains 810 amino acids, from residue 71 to 398 and 467 to 949.

The overall structure of FKP exhibits a tetrameric organization. It is about 190 Å in height and 110 Å in width (Fig. 1D). Each monomer is composed of an N-terminal GDP-fucose pyrophosphorylase domain, a C-terminal fucokinase domain and a linker in between (Fig. 1E). The GDP-fucose pyrophosphorylase domain contains a typical Rossmann fold and a left-handed β-helix fold. These two folds have been identified in a number of nucleotidyltransferases (Singh et al., 2012). In the Rossmann fold structure, a central twisted seven-stranded β-sheet is surrounded by seven α-helices. One side of the β-sheet is covered by three α-helices and the other is covered by four α-helices. A twisted β-sheet hairpin structure was identified on the three α-helices side. The fucokinase domain of FKP, as predicted, adopts a GHMP sugar kinase fold (Fig. 1E). Fucokinase belongs to the GHMP kinase family, which includes galactokinase, homoserine kinase, mevalonate kinase and phosphomevalonate kinase (Kotake et al., 2008). The linker between NTD and CTD of FKP is formed of 5 α-helices, which may be structurally rigid.

The tetrameric organization of FKP is mediated mainly by the CTDs. Two interfaces are identified in the structure, with one interface formed by a six-stranded β sheet interaction and the other by a loop-helix interaction (Fig. 1F, left panel). The β strands interface contains three core residues from each subunit, Q623, Q627 and Y629. Q623 from two close subunits may form two hydrogen bonds between each other, and Q627 possibly forms a hydrogen bond with Y629 from another subunit in the structure model (Fig. 1F, top panel on the right, and Fig. S3A). The loop-helix interface is mediated by a loop between two β-sheets from 786 to 804, and the C-terminus of an α-helix. R787, W788, H792 and Q851 are involved in this interface (Fig. 1F, bottom panel on the right, and Fig. S3B).

We applied the NADH coupled assay and the pyrophosphate assay to characterize the fucokinase activity and the GDP-fucose pyrophosphorylase activity of FKP, respectively. The *K*_m_ and *K*_cat_ of FKP fucokinase activity were determined to be 66.29 ± 1.36 μmol/L and 0.39 ± 0.003 s^−1^, respectively. And the *K*_m_ and *K*_cat_ of FKP GDP-fucose pyrophosphoralyse activity were verified to be 32.95 ± 3.70 μmol/L and 10.07 ± 0.41 s^−1^, correspondingly, which is consistent with previous report (Wang et al., 2009).

Sequence alignments between FKPs in different species and the human fucose-1-phosphate guanylyltransferase suggest that residues from 73 to 89 in BfFKP are highly conserved (Fig. S2A). These residues locate in a loop region between the Rossmann fold and the left-handed β-helix fold (Fig. 2A, top panel on the left). The primary sequence of this region contains a HXGGXSXRXP(X)_5_GK motif, where X represents any amino acid (Fig. S2A). We carried out mutagenesis analysis of residues in this region to find out whether they are related to the nucleotide transfer. The result shows that the GDP-fucose pyrophosphorylase activities for H73A, G75A and P82A mutants are greatly reduced. G76A, R80A, G88A and K89A mutants almost completely lose their GDP-fucose pyrophosphorylase activities (Fig. 2B, top panel). Taken together, it strongly suggests that this loop region is critical to the GDP-fucose pyrophosphorylase activity of FKP and may be the putative GTP binding site during catalysis.

**Figure 2:**
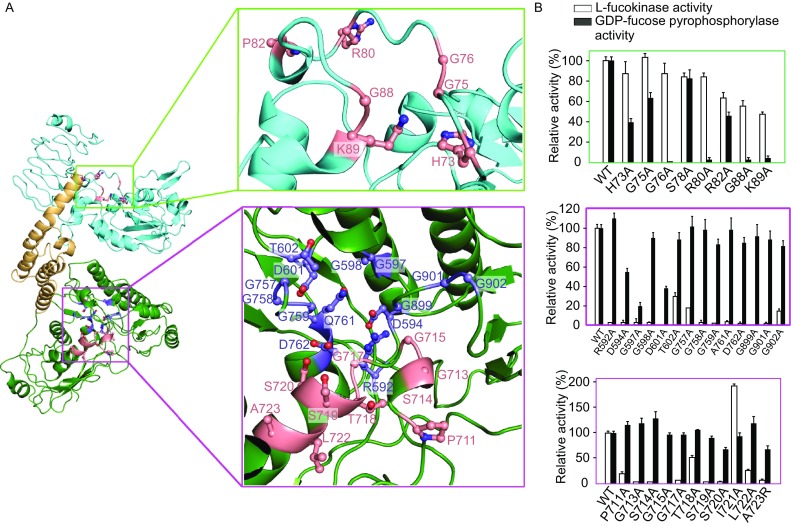
**Catalytic sites in FKP and functional analysis of FKP**. (A) Catalytic sites in FKP. Two close-up views of the active sites are shown on the right. The GTP binding sites are colored in salmon on the top panel. The fucose binding sites are colored in blue and the ATP binding sites are colored in salmon on the bottom panel. (B) The single point mutation reduced the activities of FKP, fucokinase activity or GDP-fucose pyrophosphorylase activity. The relative activity of each reaction was normalized to that of the reaction containing substrate and FKP (100%). Each reaction was repeated at least three times. Error bars represent s.e.m

Meanwhile, sequence alignment of the fucokinase family also reveals high sequence similarities (Fig. S2B). A similar motif likely to be the ATP-binding site (PXGSGLGTSSILA, residues 711 to 723) according to previous studies of galactokinase (Hartley et al., 2004) and three other glycine-rich motifs (GGWXDTPP, residues 597 to 604; TGGGWQDQ, residues 756 to 763; GAGGGG, residues 899 to 904) are identified. In our structure, these four motifs form a large pocket (Fig. 2A, bottom panel on the left). To assess the function of these motifs, we generated a series of point mutations and examined their fucokinase activities. For the ATP-binding motif, all of the mutants except I721A have reduced fucokinase activity (Fig. 2B, bottom panel). For mutants with G713A, S714A, G715A, G717A, S719A, S720A and A723R, the fucokinase activities are totally abolished. For the glycine-rich motifs, result shows that the T602A, G757A and G902A mutants have greatly reduced fucokinase activities, while mutants with R592A, D594A, G597A, G598A, D601A, G758A, G759A, Q761A, D762A, G899A and G901A, respectively, retain hardly any fucokinase activities (Fig. 2B, middle panel). This suggests that this large pocket surrounded by the highly conserved residues is a potential substrate-binding site in the fucokinase domain of FKP.

The structure of *B*. *fragilis* FKP reported here provides valuable information about the molecular basis for substrate recognition and the catalysis mechanism. As reported, a chemoenzymatic method has been illustrated which offers a practical and versatile approach for the synthesis of GDP-fucose and the Le^x^ trisaccharide glycan by *B*. *fragilis* FKP (Wang et al., 2009). Therefore, the structural and biochemical investigation of FKP will be prominently beneficial for GDP-fucose synthesis and industrial applications in large-scale production of fucose conjugates.

## Electronic supplementary material

Below is the link to the electronic supplementary material.

Electronic supplementary material 1 (PDF 1089 kb)
