# Supplementary material for: Cryo-EM structure of L-fucokinase/GDP-fucose pyrophosphorylase (FKP) in Bacteroides fragilis
Source: Protein Cell. 2018 Sep 21;10(5):365–9. doi: 10.1007/s13238-018-0576-x (PMC6468028; doi:10.1007/s13238-018-0576-x)
Supplement: Supplementary file 1 — Electronic supplementary material 1 (PDF 1089 kb) [file 13238_2018_576_MOESM1_ESM.pdf]

Supplementary Information

Cryo-EM Structure of L-fucokinase/GDP-fucose pyrophosphorylase (FKP) in *Bacteroides fragilis*

Ying Liu[1]*, Huifang Hu[2]*, Jia Wang[2]*, Qiang Zhou[2,5], Peng Wu[3], Nieng Yan[1,2,5], Hong-Wei Wang[1,2,4], Jia-Wei Wu[2,4], Linfeng Sun[6#]

[1]Tsinghua-Peking Joint Center for Life Sciences, School of Life Sciences, Tsinghua University, Beijing 100084, China
[2]School of Life Sciences, Tsinghua University, Beijing 100084, China
[3]Department of Molecular Medicine, The Scripps Research Institute , La Jolla, California 92037, United States of America
[4]Ministry of Education Key Laboratory of Protein Sciences, Beijing Advanced Innovation Center for Structural Biology, School of Life Sciences, Tsinghua University, Beijing 100084, China
[5]State Key Laboratory of Membrane Biology, Beijing Advanced Innovation Center for Structural Biology, School of Life Sciences, Tsinghua University, Beijing 100084, China
[6]CAS Center for Excellence in Molecular Cell Science, Hefei National Laboratory for Physical Sciences at Microscale, School of Life Sciences, University of Science and Technology of China, Hefei 230027, China

**These authors contributed equally to this work.*

[#]Correspondence: sunlf17@ustc.edu.cn. (L.S.)

[#]To whom correspondence should be addressed:

Linfeng Sun

School of Life Sciences

University of Science and Technology of China,

Hefei 230027, P.R.China

Tel: (+)-86-63606932

E-mail: sunlf17@ustc.edu.cn

Keywords: FKP, GDP-fucose, fucokinase, pyrophosphorylase, cryo-EM structure

EXPERIMENTAL PROCEDURES

Protein expression and purification

The FKP plasmids were gifts from Peng Wu's lab. The full-length FKP was subcloned into the pET21b vector (Novagen) and transformed into *Escherichia coli* BL21 (DE3) cells. Overexpression of FKP protein was induced with 0.2 mM isopropyl-β-D-thiogalactoside at an OD_{600} of 1.2. After growing for 12 h at 18 °C, cells were harvested by centrifugation, and then resuspended in lysis buffer containing 25 mM Tris-HCl, pH 8.0, and 150 mM NaCl. After sonication and centrifugation, the supernatant was loaded onto to Ni^{2+} affinity resin (Ni-NTA, Qiagen) and washed with buffer containing 25 mM Tris-HCl, pH 8.0, 150 mM NaCl, and 30 mM imidazole. The target protein was eluted from the Ni^{2+} affinity resin with buffer containing 25 mM Tris-HCl, pH 8.0, 150 mM NaCl, and 500 mM imidazole. The elution was further fractionated by ion-exchange chromatography (Source 15Q, GE Healthcare) and purified by size exclusion chromatography (Superdex-200, GE Healthcare) in buffer containing 25 mM Tris-HCl, pH 8.0, and 150 mM NaCl. The peak fractions were collected, flash-frozen in liquid nitrogen and stored at −80 °C for later use.

All FKP point mutants were generated by PCR-based method. The wild-type and mutant FKP proteins were individually purified as previously described and used for enzymatic activity analysis.

Analytical ultracentrifugation analysis

Analytical ultracentrifugation (sedimentation velocity and sedimentation equilibrium) experiments were carried out using a Beckman XL-I analytical ultracentrifuge (Beckman Coulter) equipped with an eight-cell An-50 Ti rotor. For sedimentation velocity, absorbance at 280 nm versus radial location was recorded during centrifugation at 50 000 rpm at 20 °C. For sedimentation equilibrium, data were collected by UV detector at 4 °C for three protein concentrations (OD_{280} = 0.2, 0.4 and 0.6) at three rotor speeds (8 500, 13 000 and 18 000 rpm). The data of sedimentation velocity and sedimentation equilibrium were analyzed using Sedfit and Sedphat (Schuck, 2003).

Glutaraldehyde cross-linking assay

About 0.5 mg ml^{-1} full-length FKP was mixed with glutaraldehyde at the indicated concentrations and incubated at 25 °C for the indicated time in the buffer containing 25 mM HEPES and 150 mM NaCl. Then the reaction was quenched by the addition of Tris, pH 8.0, to a final concentration of 50 mM and cross-linking result was analyzed by SDS-PAGE. The FKP protein treated by 0.025% glutaraldehyde at 25 °C for 30 min was further used for cryo-EM sample preparation.

Cryo-EM sample preparation and data acquisition

The purified FKP was diluted to a concentration of about 2 μM in a buffer containing 25 mM Tris-HCl, pH 8.0, 150 mM NaCl, 2 mM DTT immediately before preparing the frozen-hydrated grids. 4 μl of the sample was placed on a glow-discharged Quantifoil 1.2/1.3 holey carbon 400 mesh copper grids. The grid was blotted using a Vitrobot Mark VI (FEI company) using 3.0 s blotting time with 100% humidity at 22 °C, and then plunge frozen in liquid ethane cooled by liquid nitrogen. Cryo-EM data was collected on an FEI Titan Krios electron microscope, equipped with a Gatan K2 Summit direct-electron counting camera. The microscope was operated at 300 kV and images of the specimen were recorded with a defocus range of -2.0 to -3.0 μm at a calibrated magnification of 38 269 in super resolution mode of the K2 camera, yielding a pixel size of 0.65 Å. A total of 2 040 stacks with 32 sub-frames movie were recorded using the semi-automated low-dose acquisition program UCSF-Image4 (Li et al., 2015). The electron dose rate is 6.25 electrons per $Å^2$ per second and total exposure time of 8 seconds. Thus, the total accumulated dose on the specimen was about 50 electrons per $Å^2$ and each frame had an exposure time of 0.25 s.

Image processing of electron micrographs

8 183 particles were semi-automatically picked form 72 negative stain micrographs using e2boxer.py in EMAN2 (Tang et al., 2007) software package. Then the particle images were decimated twice, band-pass filtered, normalized, aligned, and classified using multivariate statistical analysis and multi-reference alignment iteratively in IMAGIC-4D (van Heel and Keegstra, 1981) software package. An initial model was generaged by EMAN2 using the best 2D class averages.

For the cryo-EM dataset, the 32 frames of each movie stack collected from the

K2 Summit camera were decimated twice, aligned and summed using the frame-based motion correction algorithm to generated a final drift-corrected micrograph for further image processing (Li et al., 2013). The contrast transfer function (CTF) parameters of each micrograph were determined using CTFFIND4 (Mindell and Grigorieff, 2003). For each data set, approximately 10,000 particles were semi-automatic picked using EMAN2 and performed reference-free 2D-classification in RELION (Scheres, 2012). The best two representative 2D class averages were selected as templates for automated particle picking in RELION. The automatic-picked particles were cleaned up by one round of 2D classification and one round of reference-based 3D-classification using RELION. The initial 3D model was low-pass filtered to 60 Å. The most homogeneous particles were selected for the final auto-refinement. The reconstruction map exhibited an overall resolution of 4.2 Å by applying D2 symmetry. It is obvious that the C-terminal of the map is rigid and N-terminal is flexible. Then we continued the 3D auto-refinement by applying a local mask around the C-terminal and the final resolution is 3.9 Å. For the more flexible N-terminal, we expanded the 96 749 particle based on the D2 symmetry, 386 996 N-terminals were rotated to the same position and classified without any alignment with a local mask. A total number of 145 674 particles were selected for C1 symmetry auto-refinement and reprocessed with additonal option “solvent_correct_fsc” by RELION 2.1. The final reconstruction of these particles markedly improved the resolution of N-terminal. Further details related to data processing are summarized in Table S1.

Model building of FKP

We use online Phyre server (Kelley et al., 2015) to obtain the initial model of FKP. Phyre gave several models based on the known crystal structures. For the residues 71-398 and 567-949, the models could be easily docked into the cryo-EM mass in chimera (Pettersen et al., 2004). For the linker between 467-566, it was built by placing the ideal alpha-helices into the density using COOT (Emsley and Cowtan, 2004). All individually fitted domains were connected subsequently using COOT. Residues 71-398, 467-566 and residues 567-949 were refined against the 4.0 Å map and 3.9 Å map separately using phenix.real_space_refine implemented in PHENIX (Adams et al., 2010). In the final FKP model, 810 residues were modeled in each molecule.

Measurement of the L-Fucokinase Activity of FKP

The L-fucokinase activity of the recombinant FKP was determined by monitoring the production of ADP using a coupled spectrophotometric assay. The assay was carried out at 25 °C in 1.8 mL reaction mixture containing 50 mM MOPS buffer, pH 7.0, 100 mM NaCl, 0.1 mM EDTA, 10 mM $MnCl_2$, 5 mM ATP, 200 μM NADH, 1 mM phospho(enol)pyruvate, 20 units/mL lactate dehydrogenase, 15 units/mL pyruvate kinase. All components except fucose were mixed in the cuvette and allowed to equilibrate for at least 5 minutes. Reactions were initiated by the addition of 5 μM L-fucose to the reaction mixture in the presence of 500 nM FKP. Progress of the reaction was monitored continuously by following the formation of NAD^+ at 340 nm, on a PerkinElmer Lambda 45 spectrophotometer, and the initial rates were determined from the linear slopes of the progress curves. The kinetic parameters (k_{cat} and K_M) were obtained by fitting the experimental data to the Michaelis-Menten equation using a nonlinear regression analysis program. The concentrations of ADP formed in FKP-catalyzed reaction were determined using an extinction coefficient for NADH of 6 220 M^{-1} cm^{-1} at 340 nm.

Measurement of the GDP-L-Fucose Pyrophosphorylase Activity of FKP

The GDP-L-Fuc pyrophosphorylase activity of the recombinant FKP was determined using a reaction mixture consisting of 50 mM MOPS buffer, pH 7.0, 100 mM NaCl, 0.1 mM EDTA, 2 mM $MgCl_2$, 200μM GTP, 0.1 mg/mL purine nucleoside phosphorylase, 200 μM 7-methyl-6-thioguanosine (MESG) and 1 unit/mL inorganic pyrophosphatase. All components except FKP and the substrate Fuc-1-P were mixed in the cuvette and allowed to equilibrate for at least 5 min. Reactions were initiated by the addition of 5 nM FKP in the presence of 5 μM Fuc-1-P. The continuous absorbance changes at 360 nm were recorded with a PerkinElmer Lambda 45 spectrophotometer, and the initial rates were determined from the linear slopes of the progress curves. The concentration of MESG was determined at 331 nm using a molar extinction coefficient of 32 000 M^{-1} cm^{-1}, and quantification of phosphate release was measured at 360 nm using the extinction coefficient of 11 200 M^{-1} cm^{-1}.

Data availability

The coordinates of the FKP have been deposited in the Worldwide Protein Data Bank

(http://www.rcsb.org) with the accession code (PDB: 5YYS). The corresponding maps have been deposited in the Electron Microscopy Data Bank (http://emdatabank.org) with the accession code (PDB: 5YYS).

References

Adams PD, Afonine PV, Bunkóczi G, Chen VB, Davis IW, Echols N, Headd JJ, Hung LW, Kapral GJ, Grosse-Kunstleve RW, McCoy AJ, Moriarty NW, Oeffner R, Read RJ, Richardson DC, Richardson JS, Terwilliger TC, Zwart PH (2010) PHENIX: a comprehensive Python-based system for macromolecular structure solution. Acta Crystallogr Sect D Biol Crystallogr *66*, 213-221.

Emsley P, Cowtan K (2004) Coot: model-building tools for molecular graphics. Acta Crystallogr Sect D Biol Crystallogr *60*, 2126-32.

Kelley LA, Mezulis S, Yates CM, Wass MN, Sternberg, M.J (2015) The Phyre2 web portal for protein modeling, prediction and analysis. Nat Protoc *10*, 845-858.

Li X, Mooney P, Zheng S, Booth CR, Braunfeld MB, Gubbens S, Agard DA, Cheng Y (2013) Electron counting and beam-induced motion correction enable near-atomic-resolution single-particle cryo-EM. Nat Methods *10*, 584-90.

Li X, Zheng S, Agard DA, Cheng Y (2015) Asynchronous data acquisition and on-the-fly analysis of dose fractionated cryoEM images by UCSFImage. J Struct Biol *192*, 174-178.

Mindell JA, Grigorieff N (2003) Accurate determination of local defocus and specimen tilt in electron microscopy. J Struct Biol *142*, 334-347.

Pettersen EF, Goddard TD, Huang CC, Couch GS, Greenblatt DM, Meng EC, Ferrin TE (2004) UCSF Chimera—a visualization system for exploratory research and analysis. J Comput Chem *25*, 1605-12.

Schuck P (2003) On the analysis of protein self-association by sedimentation velocity analytical ultracentrifugation. Anal Biochem *320*, 104-124.

Scheres SH (2012) RELION: implementation of a Bayesian approach to cryo-EM structure determination. J Struct Biol *180*, 519-30.

Tang G, Peng L, Baldwin PR, Mann DS, Jiang W, Rees I, Ludtke SJ (2007) EMAN2: an extensible image processing suite for electron microscopy. J Struct Biol *157*, 38-46.

van Heel M, Keegstra W (1981) IMAGIC: a fast, flexible and friendly image analysis software system. Ultramicroscopy *7*, 113-129.

Supplementary Table S1

The EM maps and PDB information of BfFKP

EM maps

	#1 (EMD-6859)	#2 (EMD-6860)	#3 (EMD-6861)
**Data Collection**			
Micrographs	2,040	2,040	2,040
Particles	96,749	96,749	145,674
Pixel size (Å)	0.65	0.65	0.65
Defocus range (μm)	2-3	2-3	2-3
Electron dose ($e^{-}Å^{-2}$)	50	50	50
**Refinement**			
Resolution (Å)	4.2	3.9	4.0
B-factor ($Å^{2}$)	-135	-135	-135

PDB (PDB-5YYS)

**Model composition**	
Non-hydrogen atoms	6,016 * 4
Protein residues	810 * 4
**RMS deviations**	
Bonds (Å)	0.001
Angles (°)	1.86
**Ramachandran plot**	
Most favored (%)	65.9
Additional allowed (%)	32.5
Generously allowed (%)	1.6
Disallowed (%)	0

Supplementary Figure S1

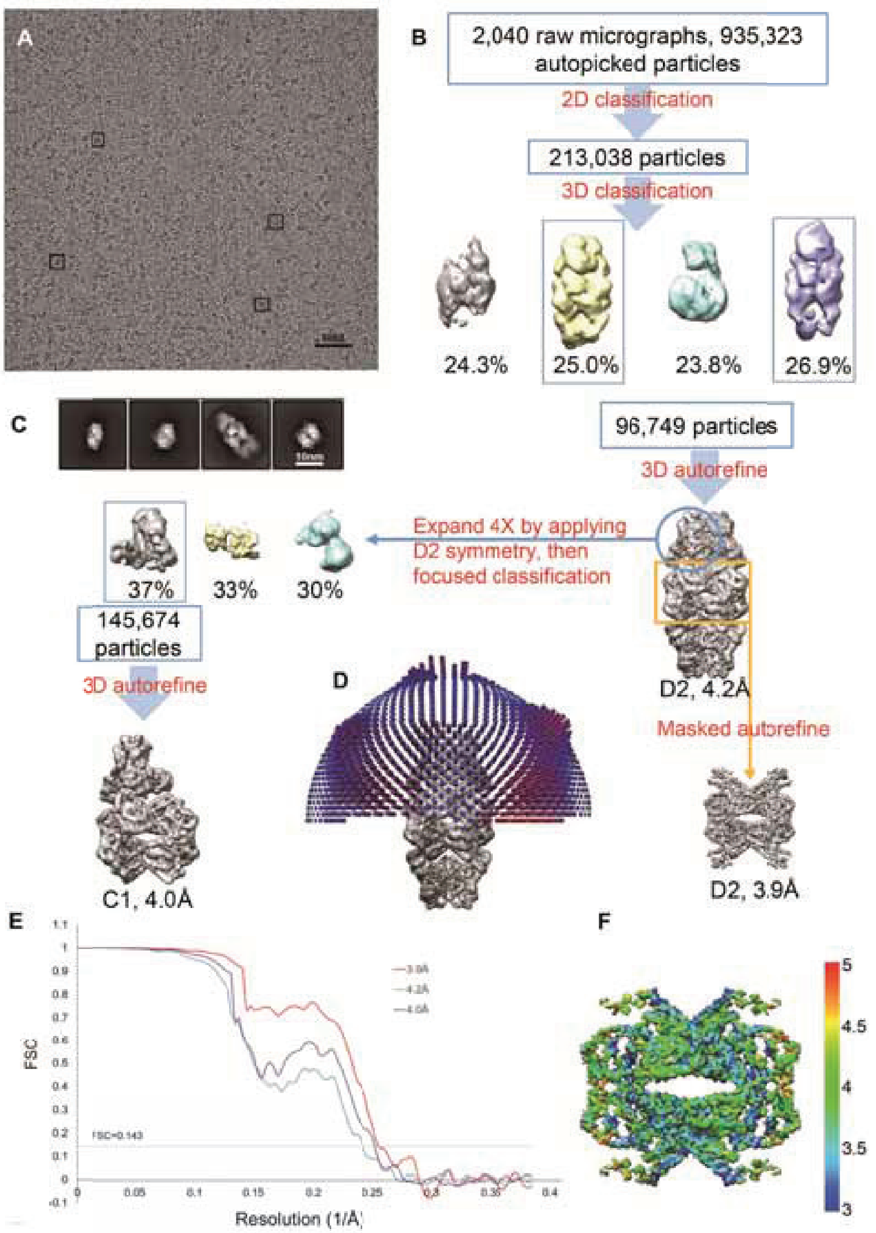

Fig. S1 Cryo-EM analysis of wild type FKP.

(A) A representative area from a raw micrograph of the frozen-hydrated FKP complex taken under the 300 kV Titan Krios microscope. Typical particles are marked by square boxes. (B) Schematic outline of the image processing steps used to obtain the final cryo-EM reconstructions of the FKP complex. (C) Typical 2D class averages of the complex. (D) Angular distribution of particles for the 4.2 Å 3D reconstruction. (E) Corrected Fourier Shell Correlation (FSC) curves of the 3D reconstructions in RELION. (F) Local resolution map of the final 3.9 Å 3D reconstruction estimated using RESMAP.

Supplementary Figure S2

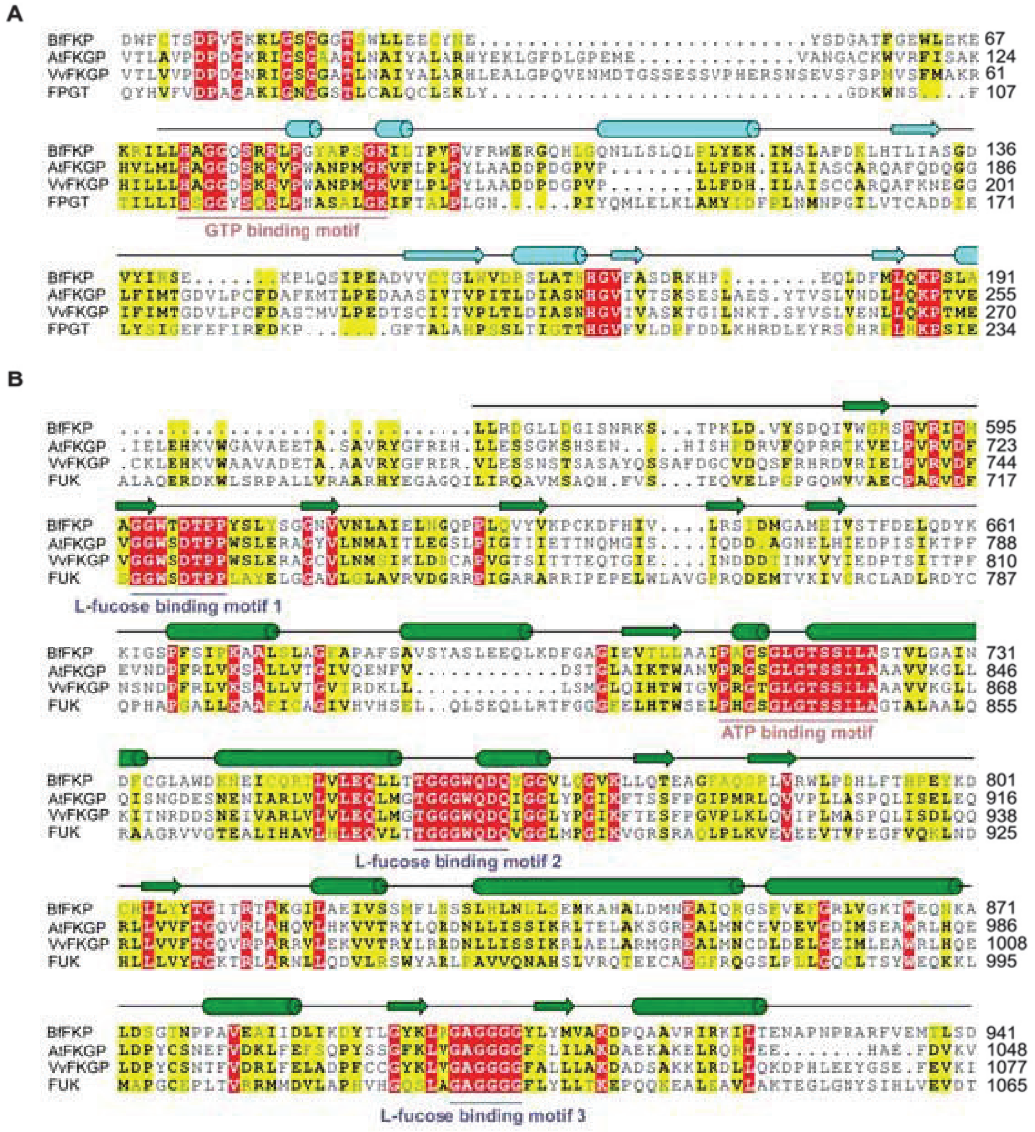

Fig. S2 Multiple sequence alignment of bifunctional fucokinase/GDP-fucose pyrophosphorylase from different species and isoenzymes from human using ClustalW. Secondary structural elements of BfFKP are indicated above the sequence alignment and color-coded consistent with the color scheme for the protomer structure. Invariant amino acids are shaded red and conserved residues are colored yellow. (A) Sequence alignment for GDP-fucose pyrophosphorylase. (B) Sequence alignment for fucokinase. The Uniprot IDs for the aligned sequences are: BfFKP (Bifunctional fucokinase/GDP-fucose pyrophosphorylase from *Bacteroides fragilis*): Q58T34; AtFKGP (Bifunctional fucokinase/fucose pyrophosphorylase from *Arabidopsis thaliana*): Q9LNJ9; VvFKGP (Bifunctional fucokinase/fucose pyrophosphorylase from *Vitis vinifera*): D7UCW9; FPGT (fucose-1-phosphate guanylyltransferase from human): O14772, and FUK (L-fucose kinase from human): Q8N0W3.

Supplementary Figure S3

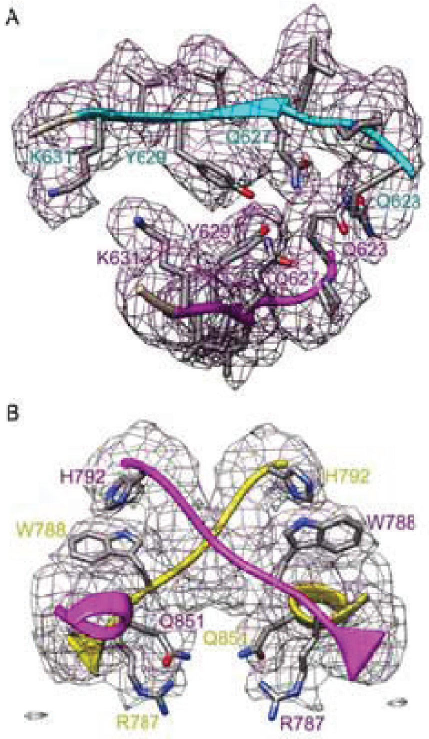

Fig. S3 EM density maps for representative segments of FKP tetramer interfaces.

(A) The EM density maps for the β strands interface. (B) The EM density map for the loop-helix interface. Bulky residues that were used to facilitate sequence assignment during model building are labeled. The side groups of representative bulky residues are shown. The maps were prepared using Chimera.
